# The usefulness of appetite and energy intake-based algorithms to assess treatment effect of a bacterial infection: An observational prospective study

**DOI:** 10.1371/journal.pone.0186514

**Published:** 2017-10-26

**Authors:** Viktor Peny, Fredrik Månsson, Fredrik Resman, Jonas Ahl, Johan Tham

**Affiliations:** Infectious Diseases Research Unit, Department of Clinical Sciences, Lund University, Malmö, Sweden; National Yang-Ming University, TAIWAN

## Abstract

**Background:**

The diagnosis of infectious diseases and the duration of antibiotic therapies are generally based on empirical rules. Studies implicate that the use biological markers can be used as a reliable method to shorten antibiotic therapies. The return of appetite is a clinical aspect of recovery from an infection that may be used to guide antibiotic therapies.

**Objective:**

To compare changes in appetite and daily energy intake with changes in CRP-levels in patients recovering from an infection.

**Design:**

Observational study using a consecutive sample of patients admitted to the unit for infectious diseases at a University Hospital in Sweden, February to April 2014. Energy intake, CRP-levels and appetite were recorded daily. Energy intake was calculated using estimated energy contents. Appetite was measured using a validated visual analogue scale. Changes in daily energy intakes, CRP-levels and appetite were analysed.

**Results:**

49 patients (51% men) were included in the analysis from the overall population of 256 patients. During the length of the stay (median 3 days) CRP-levels fell in 92% of the patients (*p*<0.001), daily energy intake increased in 73% (median intake +6381 kJ/day, *p*<0.001) and appetite increased in 55% of the patients (*p* = 0.181). VAS-estimations of appetite augmented in 55%, decreased in 41% and were equal in 5% of the patients (*p* = 0.181). There was a non-significant difference in the within-subject variances in daily energy intake between female and male patients but not in other subsets.

**Conclusions:**

We found a significantly increase in the daily energy intake but not in self-estimated appetite in patients recovering from an infection. We suggest measuring the daily energy intake as a complement to other biological and clinical markers among inpatients to assess treatment effect.

## Introduction

Human appetite is controlled by neuroendocrine hormones. Ghrelin, often referred to as the ‘hunger hormone’, is released to the circulation by gastric cells and increases both feeling of hunger and food intake. Other hormones, such as Peptide YY have opposite effects. Peptide YY is released after food intake by enteroendocrine cells in the distal gastrointestinal-tract and induces the feeling of satiety and thereby terminates the meal [1–3].

Loss of appetite and reduced nutrient intake are parts of the symptomatology presented in infected patients together with fever, headache, myalgia and malaise [4]. The decreased nutrient intake and appetite are considered as beneficial to the host [5,6] and their causes have been studied in numerous animal models. When administered parentally, bacterial antigens (such as lipopolysaccharide and muramyl diptide) reduce nutrient intake in mice [7]. This reaction is considered to be mediated by cytokines (e.g. interleukin-1 and 6, tumor necrosis factor α and interferon-γ) excreted during the inflammatory response [8].

The diagnosis of infectious diseases and the duration of antibiotic therapies are normally based on empirical rules [9]. The duration of antibiotic therapies can be guided (and shortened) using certain biomarkers (i.e. c-reactive protein, CRP, or procalcitonin, PCT) as guides [10–12]. PCT-based algorithms have shown to be able to reduce the number of days with antibiotics by 20–23% in the intensive care unit [10,13]. There are very few studies conducted with CRP-based algorithms, but CRP appears to be equally good as PCT in guiding the duration of antibiotic therapies [11,14]. Various studies further correlate low CRP-levels at admission and rapid descent of CRP levels during the first days of antibiotic treatment with better prognosis [14–18].

We sought to investigate the possibility to assess the treatment effect of a bacterial infection by recording patients’ nutrient intake. We aimed to examine changes in appetite and energy intake during recovery from an infection. Our hypothesis was that appetite and energy intake would change in a way that they could be used to evaluate treatment effect of a bacterial infection. This could strengthen clinical instruments available to assess patients’ recovery from an infection and thereby optimizing the current use of antibiotics.

## Materials and methods

### Design and Ethics

This prospective observational study was conducted at the unit for infectious diseases at Skåne University Hospital Malmö, a Swedish tertiary hospital. Changes in appetite and energy intake were compared with CRP levels in a consecutive sample of patients. Patients were included in the study from the day of admission until discharge from the unit. Ethical approval was obtained from the Research Ethics Committee of the University of Lund, Sweden (2013/898). All patients were given written and verbal information prior to signing an informed consent form.

### Participants

The study was conducted between February 11 and April 16, 2014. All patients, 18 years old and above, admitted to the unit from the emergency department with a suspected or confirmed infectious disease were evaluated for potential inclusion. Patients excluded were as follows: patients with 1) gastroenteritis; 2) microbiological infection by fungi, parasites or virus, except Influenza A virus; 3) infections that required long-term or a specific antibiotic therapy (e.g. endocarditis, chronic osteomyelitis, infections by Mycobacterium tuberculosis, septic arthritis and prosthetic joint infection); 4) with eating disorders; 5) patients unable to receive enteral nutrition or 6) patients who stayed less than 24h at the unit. Estimations of energy intake and appetite for a minimum of 48 h were required for inclusion in analysis.

### Daily energy intake

At the unit, patients were served meals three times daily, plus drinks and snacks ad libitum. Breakfast was served at 7:30 a.m. to 8:30 a.m., lunch at 12:15 p.m. to 1:00 p.m. and dinner at 5:00 p.m. to 6:00 p.m. The energy content of the meals served was standardized to 2100 kJ at lunch and 1700 kJ at dinner. The patients themselves decided the composition of their breakfasts.

Patients’ food intake were recorded by an observer and their daily energy intake was calculated using estimated energy contents. Observer-recorded energy intake designs may underestimate patients energy intakes compared to calorimetric analyses of duplicates of patients alimentary intake [19,20]. Yet, this method was chosen, as it was more suitable for clinical use. Energy contents were estimated using the Swedish Food Composition Database maintained by Swedish National Food Agency [21]. The version used in this study, version 2014-01-28, contains data for approximately 2100 food items and dishes. Analyses to estimate nutrient values are carried out by L Swedish National Food Agency, food industries and food trade organisations. For each patient the resting energy expenditure was calculated according to Nordic Nutrition Recommendations 2012 [22], and compared to his or her energy intake at the day of discharge.

### Appetite-estimation

Quotidian estimations of patients’ appetite before lunch were performed using a visual analogue scale, VAS. The VAS was composed of a 100 mm line. Anchored at each ends were the opposites “Not at all hungry” and “I have never been more hungry”. Patients were instructed to place a vertical line across the scale corresponding to their present hunger. By measuring the distance between the mark and the left end of the line a quantification of the patient’s hunger was estimated. Patients could not compare their ratings with each other or to their previous ratings when marking the VAS. VAS-estimation has proven to be a reliable and reproducible method of measuring appetite in numerous studies [23,24].

### C-reactive protein

Plasma samples from each patient were taken and analysed daily. CRP levels were measured by immunoturbidimetry using a commercially available kit (Roche Diagnostics, Mannheim, Germany). This test has a measuring range of 0.6 mg/L–700 mg/L. The reference interval was set to <3.0 mg/L [25].

### Statistics

Categorical variables are presented using their absolute and relative frequency. Continuous data and ordinal data are presented using median and the 25–75% interquartile interval (Q1 –Q3). Data of the subjects are presented using median, minimum and maximum values.

CRP-levels and daily energy intake were asymmetrically distributed, thus considered as nonparametric continuous data. VAS-estimations were considered as ordinal data. Differences between subsets were analysed using Mann-Whitney U test and Chi-squared test as appropriate. Changes of both ordinal and nonparametric data was analysed using Wilcoxon signed rank test. 95% confidence intervals (95%CI) were derived from bootstrapped distributions [26]. Correlations between changes were performed calculating Spearman’s rank correlation coefficient (r_s_).

Missing data was considered as missing at random and was excluded from analysis. For all analysis, a two-tailed significance *p*-value <0.05 was set. All data were analysed using SPSS statistical package, version 22.0.0.1 (SPSS, Chicago, IL).

## Results

### Participants

**Fig 1:**
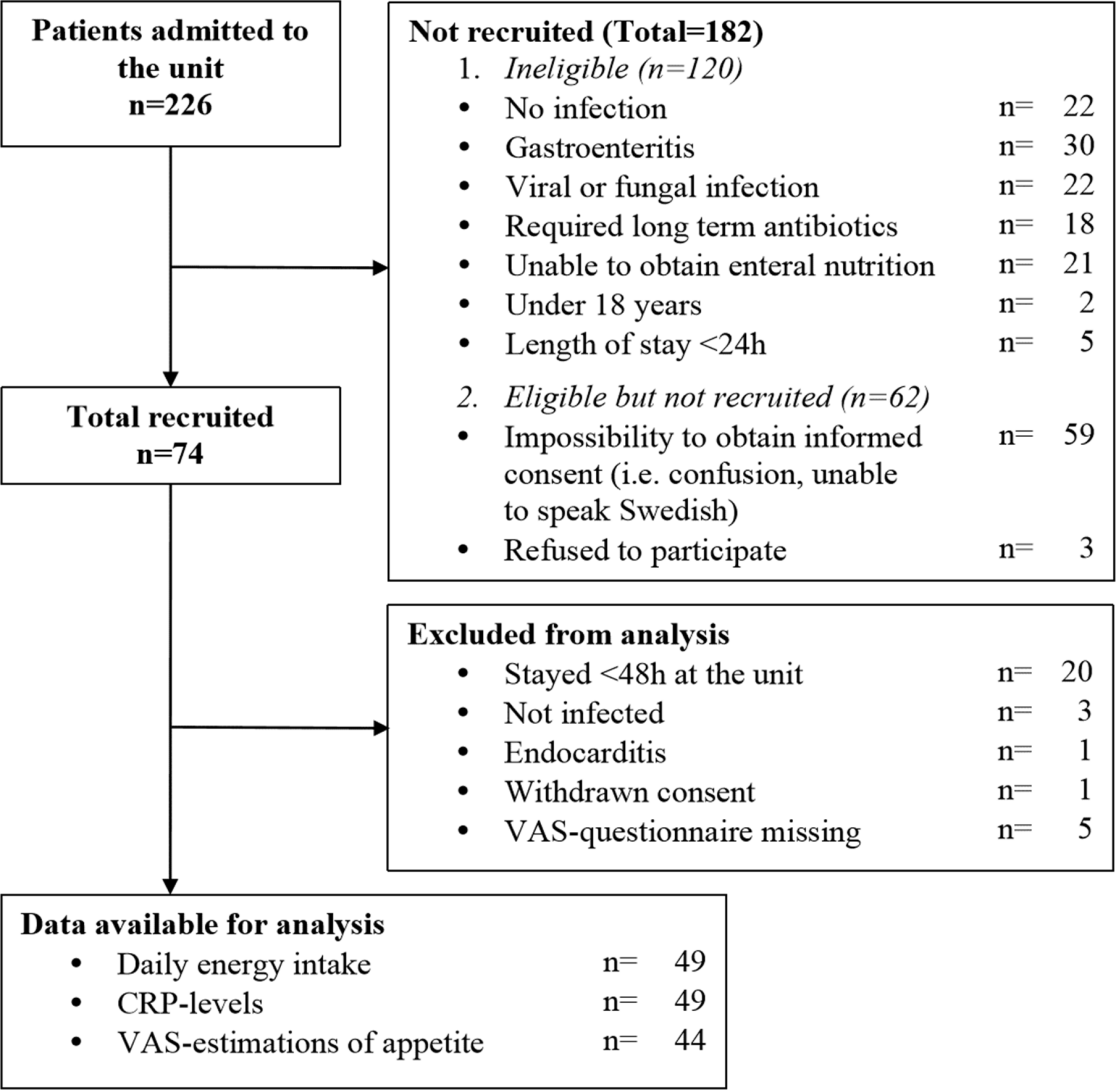
Flowchart for inclusion of patients.

During the study period 256 patients were admitted to the unit via the emergency department. Out of 130 eligible, 74 were included in the study. 25 patients were later excluded leaving 49 patients for analysis (Fig 1). VAS-questionnaires were missing for 5 patients excluding them from analysis of appetite estimations. The median age for the overall population was 65.6 (Q1 –Q3: 47.1–78.2) yrs., 54% were men. The median age for patients included in the study was 58.5 (Q1 –Q3: 42.1–77.2) yrs. and median weight was 80.0 (Q1 –Q3: 72.3–92.4) kg.

There were 16 patients (7 women and 9 men) in the age group 18–49 years, 15 patients (6 women and 9 men) in the age group 50–69 years and 18 patients (11 women and 7 men) in the age group 70–90 years (where of 3 women and 3 men older than 80 years).

The median period of inclusion in the study was 3 (Q1 –Q3: 2–5.5) days and the median time with antibiotic treatment was 10 (Q1 –Q3: 7–12) days. 12 patients were diagnosed with a malignant disease and 12 patients recieved corticosteroids parenterally or per os at some time during their hospital stay. Further characteristics of included patients are presented in Table 1.

**Table 1:** Main characteristics and outcome.

	n (%)	Days included in the study^a^	Days with antibiotics^a^	Change in CRP-levels, mg/L^a^	Change in energy intake, kJ/day^a^	Change in VAS estimation of appetite
***Sex***						
**Women**	24 (49%)	3.5 (2–12)	8 (2–23)	-105 (-353–53)	1234 (-962–3410)	0 (-53–67)
**Men**	25 (51%)	3 (2–10)	11 (1–28)	-46 (-211–56)	1569 (-5000–4121)	3 (-41–60)
***Age groups***						
**18–49**	16 (33%)	2.5 (2–10)	7.5 (2–28)	-27.5 (-347–53)	1538 (-5000–3410)	2 (-33–59)
**50–69**	15 (31%)	3 (2–10)	11 (7–18)	-101 (-353–56)	879 (-2218–4121)	3 (23–60)
**70–88**	18 (37%)	4.5 (2–12)	9 (1–23)	- 105 (-290–1)	1559 (-1966–3891)	0 (-53–67)
***Patients treated with corticosteroids***						
**Yes**	12 (25%)	3.5 (2–12)	9 (2–17)	-105 (-347 –-21)	1736 (-962–3410)	1 (-8–67)
**No**	37 (75%)	3 (2–11)	11 (1–28)	-66 (-353–56)	1192 (-5000–4121)	0.5 (-53–60)
***Patients diagnosed with cancer***						
**Yes**	12 (25%)	6 (2–10)	11 (2–18)	-56 (-353–14)	1851 (-1159–4121)	0 (-53–60)
**No**	37 (75%)	3 (2–12)	10 (1–28)	-66 (-347–56)	1276 (-5000–3410)	1 (-41–67)
***Primary Diagnosis***						
**Pneumonia**	24 (49%)	3 (2–12)	10 (2–28)	-115.5 (-353–56)	1096 (-5000–3891)	1 (-19–67)
**Pyelonephritis**	10 (20%)	2.5 (2–11)	8 (6–12)	-34 (-290 –-15)	1015 (-293–3138)	-12 (-37–30)
**Skin and Soft Tissue Infection**	5 (10%)	3 (2–5)	13 (7–15)	-21 (-165–1)	1569 (-167–3410)	1.5 (-32–10)
**Neutropenic Fever**	4 (8%)	4 (3–10)	11.5 (11–15)	-36 (-138–14)	2761 (-1159–4121)	11 (-33–60)
**Influenza**	3 (6%)	5 (2–6)	2 (1–2)	-20 (-66–1)	1757 (-1966–3243)	-41 (-53–59)
**Other**	3 (6%)	9 (4–9)	11 (5–23)	-158 (-347 –-24)	1527 (-794–2842)	17 (0–49)
***Microbiological Diagnosis***						
**Escherichia coli**	9 (18%)	3 (2–11)	8 (7–12)	-27 (-290–14)	1192 (-292–3138)	-12 (-37–30)
**Streptococcus pneumoniae**	6 (12%)	4 (2–10)	9.5 (7–15)	-129.5 (-211–53)	2186 (209–3159)	17 (-8–30)
**Influenza A virus**	2 (4%)	3.5 (2–5)	1.5 (1–2)	-9.5 (-20–1)	-105 (-1966–1757)	- 47 (-53–41)
**Staphylococcus aureus**	2 (4%)	2 (2–2)	11.5 (9–14)	-22.5 (-46–1)	1600 (1548–1653)	-32 (-32–32)
**β-hemolytic Streptococci**	2 (4%)	3 (3–3)	11 (7–15)	-159 (-165 –-153)	826 (-167–1820)	3.5 (1–6)
**Other**	5 (10%)	7 (2–9)	18 (11–28)	-158 (-353–14)	1569 (-795–3891)	-0.5 (-18–38)
**Unknown Agent**	23 (47%)	4 (2–12)	10 (2–17)	-66 (-347–56)	899 (-5000–4121)	10 (-28–67)
*Overall*	-	**3 (2–5.5)**^**b**^	**10 (7–12)**^**b**^	**-66 (-116.5 –-41)**^**b**^	**1527 (785–1705)** ^**b**^	**1 (-5–15)**

^a^ Median (min–max)

^b^ median (95%CI)

### Primary outcome

**Fig 2:**
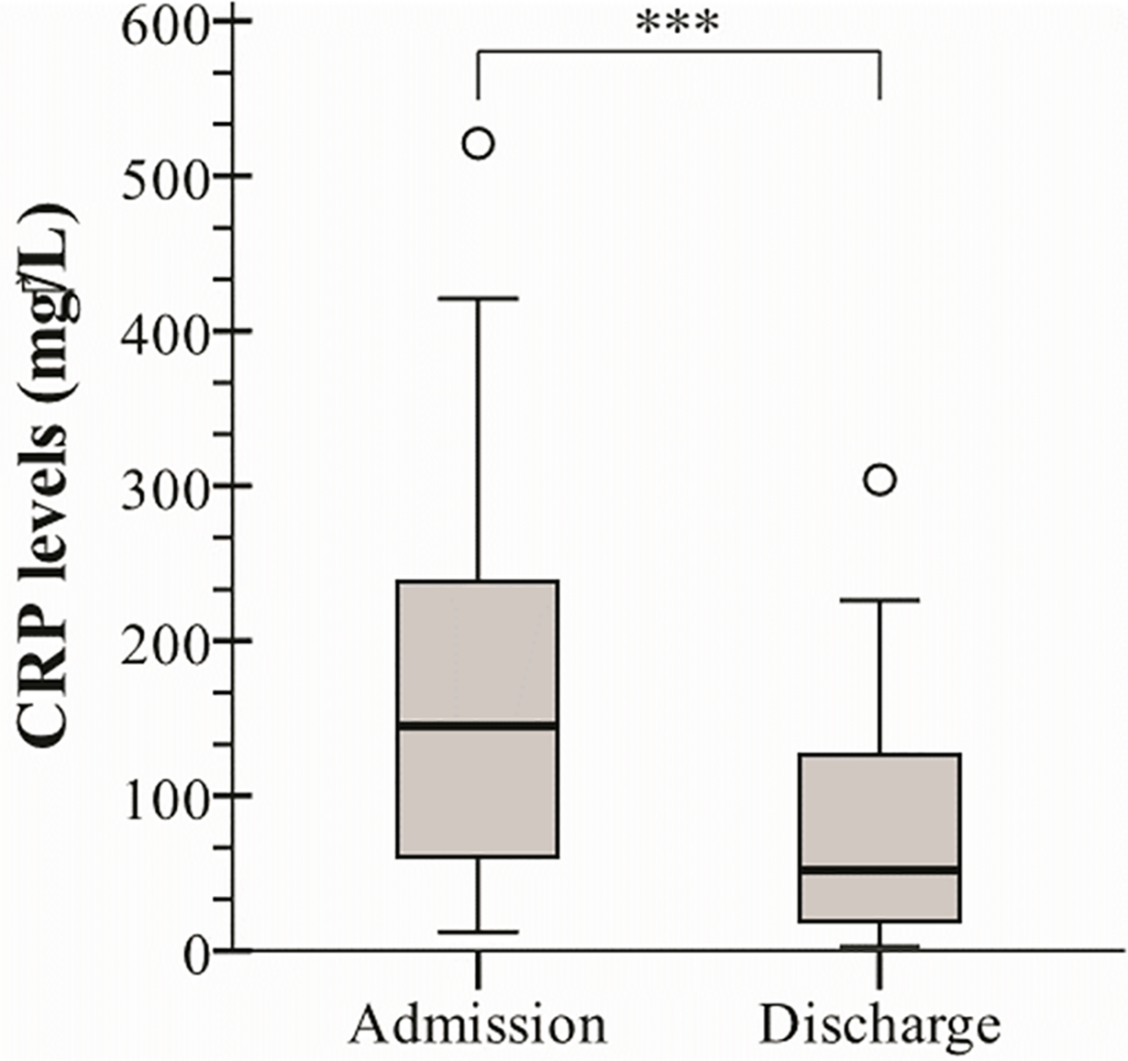
C-reactive protein (CRP) levels at the day of admission and discharge from the unit. The bottom and top of the box represents the first and third quartile. The line within the box represents the median, and the vertical lines above and below the box signify the maximum and minimum values still within 1,5 interquartile range. Outliers are represented by a o. *** = *p*<0.001 (Wilcoxon signed rank test).

At admission CRP levels were elevated in all patients, median 145 (Q1 –Q3: 59.5–242.5) mg/L. CRP was at discharge lowered in 92% of the patients, median 52 (Q1 –Q3: 19–127) mg/L (*p*<0.001). Female patients presented a higher median CRP level at admission (203 mg/L) than male patients (93 mg/L) (*p*<0.05*)*. The median change in CRP levels was –105 mg/L in female patients and –46 mg/L in male patients comparing levels at admission and discharge (*p*<0.05) (Fig 2).

**Fig 3:**
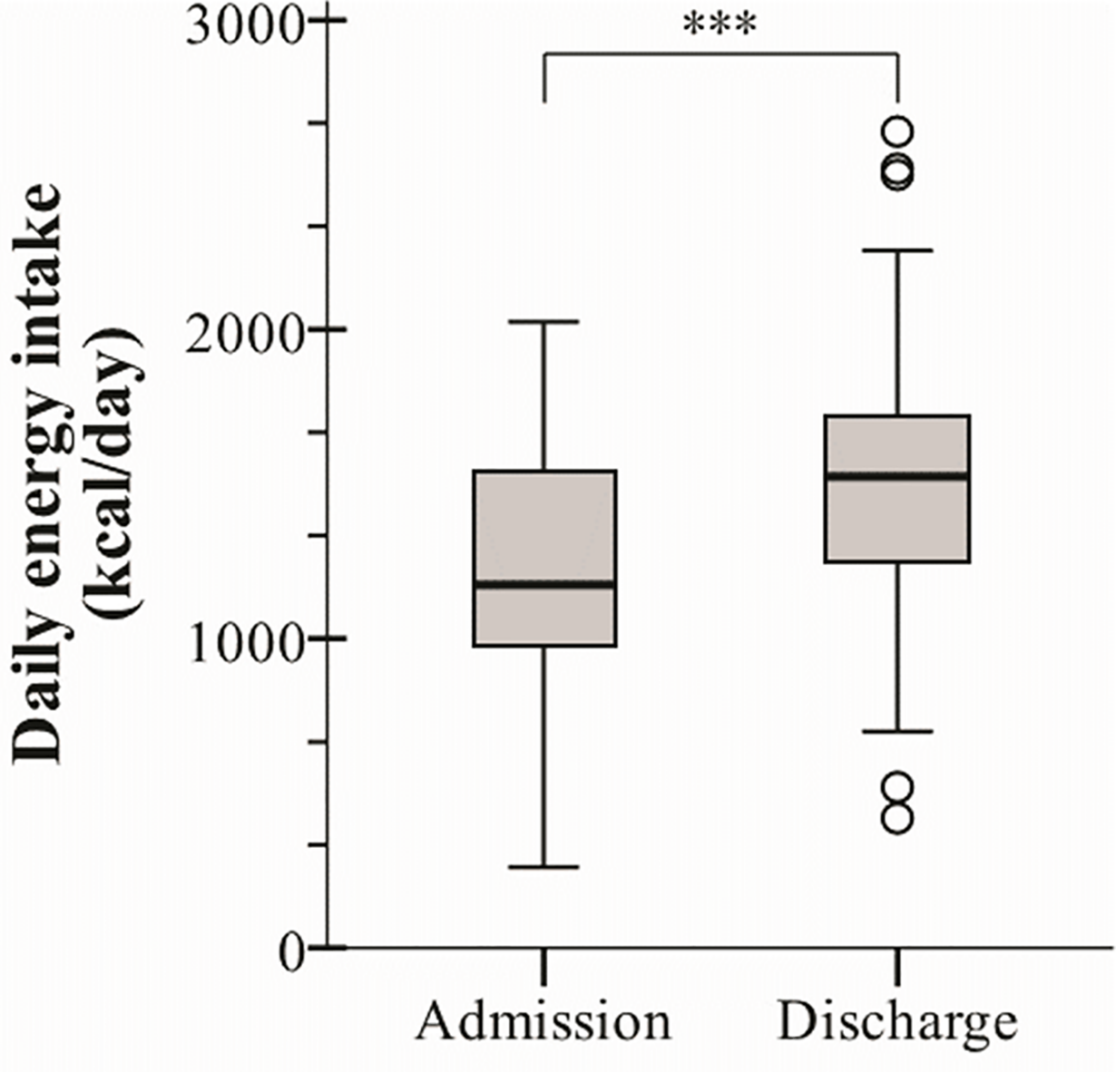
Daily energy intakes at the day of admission and discharge from the unit. The bottom and top of the box represents the first and third quartile. The line within the box represents the median, and the vertical lines above and below the box signify the maximum and minimum values still within 1,5 interquartile range. Outliers are represented by a o. *** = *p*<0.001 (Wilcoxon signed rank test).

The median daily energy intake at admission was 4916 (Q1 –Q3: 4090–6464) kJ/day compared with 6381 (Q1 –Q3: 5167–7207) kJ/day at discharge, increasing in 73% of the sample. The median daily increase was 1527 (95%CI: 785–1705) kJ/day (*p*<0.001), representing a median daily increase of 28.9% (95%CI: 14.1–34.9%) (Fig 3). Nevertheless, the changes in energy intakes did not correlate with the changes in CRP levels (r_s_ = -0.153, *p* = 0.293). Some subset of subjects (i.e. patients with cancer, corticosteroid treatment or infected with *S*. *pneumoniae*) did increase more in energy intake than their counterparts (Table 1). The daily energy intake at discharge was lower than the estimated resting energy expenditure in 57% of the patients; median difference -244 (Q1 –Q3: -2215–1110) kJ/day.

**Fig 4:**
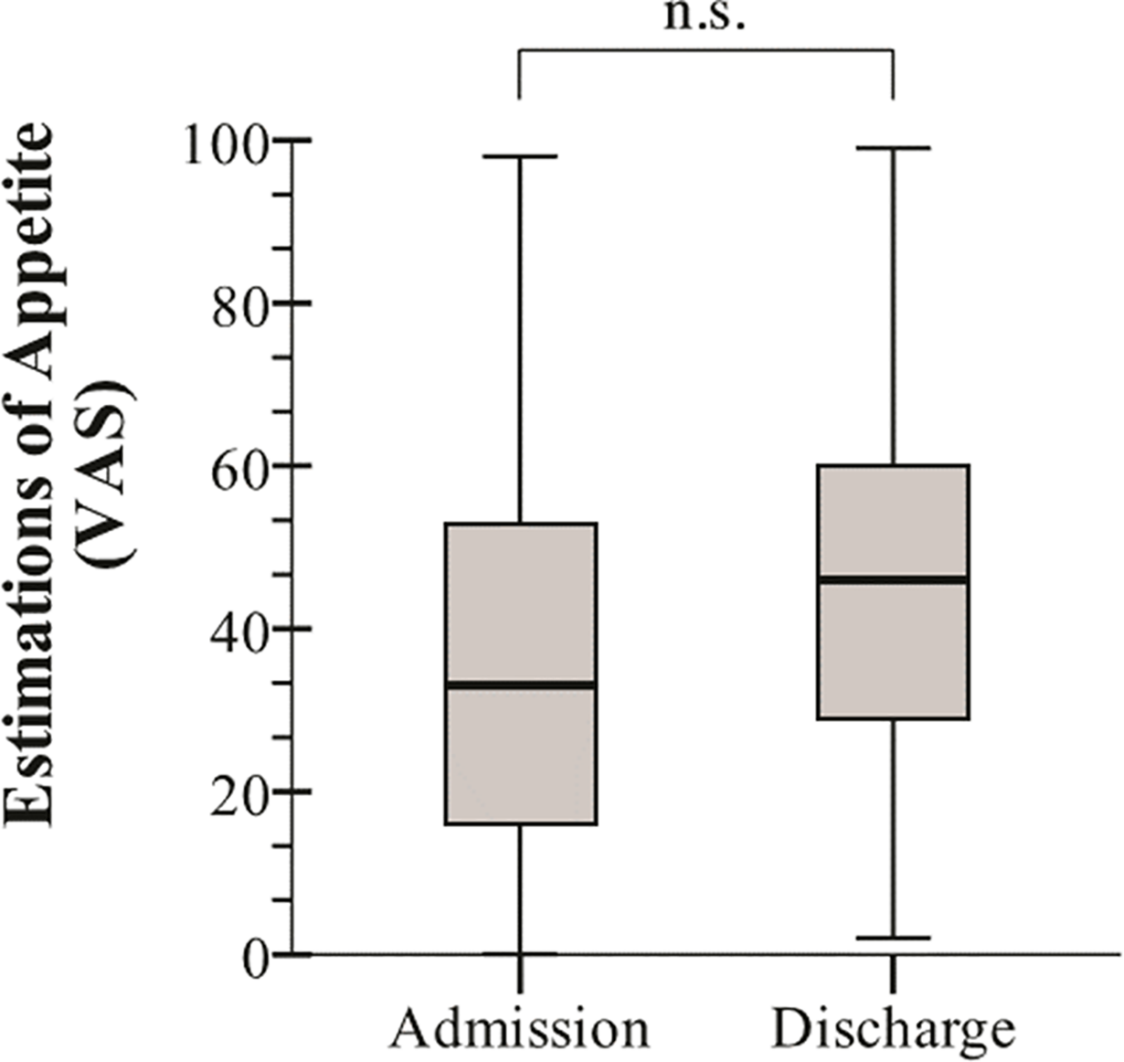
Self-estimated appetite using a visual analogue scale (VAS) at the day of admission and discharge from the unit. The bottom and top of the box represents the first and third quartile. The line within the box represents the median, and the vertical lines above and below the box signify the maximum and minimum values. NS = not significant (*p* = 0.181, Wilcoxon signed rank test).

VAS-estimations of appetite augmented in 55%, decreased in 41% and were equal in 5% of the patients (*p* = 0.181). The median VAS-estimation of appetite at admission was 33 (Q1 –Q3: 15.75–53.5) and at discharge 46 (Q1 –Q3: 27.75–62.5) (Fig 4). The changes in appetite neither correlated to CRP levels (r_s_ = -0.30, *p* = 0.719) nor to energy intake (r_s_ = 0.100, *p* = 0.211).

After an initial peak at the first two days of inclusion, CRP levels fell progressively in 84% of the patients, of which 53 percentage points decreased progressively from the first day of inclusion. Due to a within-subject variance, the same pattern was not presented in the daily energy intakes. Only 43% of the patients increased their energy intake progressively, of which 29 percentage points increased progressively from the first day of inclusion. Male patients tended to increase in their daily energy intake more progressively than female patients. 56% of the male patients and 29% of the female patients increased in their energy intake progressively (*p* = 0.058). The within-subject variance in daily energy intake was however present regardless of patients’ age, weight, infection focus and in patients with or without cancer, on-going chemotherapy or corticosteroid treatment.

There was no difference in the median change in energy intake comparing patients re-admitted within 28 days after discharge for inpatient care of an infectious disease (n = 7, +1192 kJ) and patient not re-admitted within 28 days (n = 42, +1537 kJ) (*p* = 0.886). The median change in CRP of patients re-admitted was -42 mg/L whilst the median change of patient not re-admitted was -69.5 mg/L (*p* = 0.432).

In addition to the univariate analyses, linear regression analyses for the outcomes of change in CRP levels, energy intake and VAS estimations of appetite, adjusted for age, gender and primary diagnosis were performed. There were no significant levels of change in any of these parameters.

## Discussion

We found that comparing the day of admission with the day of discharge there was a significant increase in energy intake but not self-estimated appetite. The increase in energy intake should be considered as clinically relevant since it corresponded to more than a quarter of the daily energy intake at admission. However, at an individual level the daily energy intakes revealed an abundant within-subject variance, obscuring the changes seen at a group level.

Certain limitations should be considered. First, the observer-recorded method used for estimating energy intake may underestimate patients’ daily energy intakes compared to more accurate models. Supporting the presence of this suggested error is the negative difference between each patient’s daily energy intake at discharge and his or her estimated resting energy expenditure. This could be caused by both an underestimation of patients’ energy intake as well as patients being discharged prior to full recovery. Still, this study aimed to identify new clinical markers of recovery from infection; hence we chose the method that we considered to be the most clinically applicable despite its potential underestimation.

Second, both patients with cancer or corticosteroid treatment were included in the study. These patients presented amplified increases in energy intake. Due to the limited patient sample, it was not useful to perform accurate multivariate analyses of potential factors affecting energy intake (e.g. cancer, corticosteroids, microbiological diagnosis). However, the energy intake increased even in patients without cancer or corticosteroid treatment, suggesting that recovery from infection in itself do increase a patient’s energy intake. Third, 27% of the patients did not increase their energy intake. This may indicate that our results are misleading but can also have been caused by within-subject variances without affecting the conclusion that energy intake increases in patients recovering from an infection. The results should also be interpreted knowing that the CRP is a supportive criterion used for discharge from the unit. This could result in an overestimation of the significance of the changes in CRP-levels.

This is to our knowledge the first study investigating whether appetite and energy intake can be used as markers for recovery from an infection; thus the results should be interpreted with caution. Although the results indicate significantly increasing energy intakes in patients recovering from an infection, we would not recommend using daily energy intake single-handedly as a marker of recovery, due to the abundant within-subject variance. It is plausible that the inflammatory reaction is only one of several factors affecting patients' appetite and energy intake, reducing its potential specificity as a marker of infection. Changes in energy intake may rather be used as complement to other clinical and biological markers in order to strengthen the assessment of patients’ recovery. In comparison, CRP levels are better suited as markers of recovery since they fell progressively in a vast majority of patients. It is still possible that larger studies could be used to develop energy intake based algorithms to guiding antibiotic therapies. Such an algorithm may have advantages in costs and simplicity to current existing algorithms.

As for the VAS-estimation of appetite, it appears to be a poor method for assessing recovery from an infection. The changes in estimates of appetite were neither significant nor did they correlate to changes in CRP-levels and daily energy intakes, leading to the assumption that visual analogue scales may not be a good way of measuring appetite in infected subjects.

The study sample corresponds well to the variety of ages, gender, infection foci likely to be found at a unit for infectious diseases at a Swedish tertiary hospital, Yet, considering the study size and that the majority of patients admitted to the unit were not included we urge for more studies to be carried out in this field prior to using energy intake as a marker of recovery. Further studies are also needed to better estimate the true decrease in energy intake in infected patients.

### Conclusions

This study indicates that daily energy intakes increase in patients recovering from an infection, most likely due to a reduced energy intake during the antecedent infection. The same does not apply to VAS-measured appetite, in which within-subject variances concealed any potential difference. Similar, the changes in energy intake are obscured by within-subject variances but only at an individual level. For this reason, changes in daily energy intake could be a valuable complement to other biological and clinical markers among inpatients suffering from infectious diseases. There is a need to discuss optimal ways to reliable estimate appetite before a full scale study on the subject will be conducted.

## Supporting information

S1 Fig File of raw data and description of variables. (XLSX)
